# Zoonotic Bacteria and Vector-Borne Protozoa in Troglophilus Bat Colonies in Sicily (Southern Italy): A Biomolecular Survey

**DOI:** 10.3390/ani15040488

**Published:** 2025-02-09

**Authors:** Santina Di Bella, Ilenia Giacchino, Valeria Blanda, Francesca Gucciardi, Silvia Scibetta, Francesco La Russa, Antonio Lastra, Giuseppa Purpari, Rosario Grasso, Maria Teresa Spena, Bianca Maria Orlandella, Nadia Vicari, Emanuela Olivieri, Francesca Grippi, Annalisa Guercio

**Affiliations:** 1 Istituto Zooprofilattico Sperimentale della Sicilia, 90129 Palermo, Italy; santina.dibella@izssicilia.it (S.D.B.);; 2 Dipartimento di Scienze Biologiche, Geologiche e Ambientali, Università degli Studi di Catania, 95124 Catania, Italy; 3 Dipartimento di Scienze Veterinarie, Università degli Studi di Messina, 98168 Messina, Italy; 4 Centro di Referenza Nazionale per le Clamidiosi, Istituto Zooprofilattico Sperimentale della Lombardia e dell’Emilia Romagna “Bruno Ubertini”, 27100 Pavia, Italy

**Keywords:** bats, bacterial pathogens, piroplasmids, zoonosis

## Abstract

Bats, as members of the order Chiroptera, are vital to ecosystems and serve as reservoirs for numerous microorganisms, some of which can cause zoonotic diseases. Human interactions with bats are increasing due to habitat alterations, making it essential to understand their microbiota, particularly potential pathogens. This study aimed to evaluate the excretion of zoonotic bacteria and protozoa in insectivorous bats from four caves in the provinces of Ragusa, Catania, and Syracuse (Sicily, Southern Italy) using molecular biology tests for zoonotic agents, including *Bartonella henselae*, *Borrelia*, *Coxiella burnetii*, *Leptospira*, *Chlamydia*, *Rickettsia*, *Anaplasma*, and Piroplasmids. From December 2020 to April 2023, urine, fecal swabs, ocular conjunctival swabs, and oropharyngeal swabs were collected from 149 bats of six species, along with guano samples from the caves. *Bartonella henselae* DNA was detected in 3 of the 149 tested bats, one ocular conjunctival swab and two oropharyngeal swabs. *Chlamydia* spp. DNA was detected in a sample of guano, in feces, ocular conjunctival and oropharyngeal swabs of a bat, and in four urine samples. Piroplasmid DNA was detected in 10 of 149 fecal swabs and in 5 of 16 bat ectoparasites. No samples were positive for *Leptospira* spp., *Borrelia* spp., *Coxiella burnetii*, *Rickettsia* spp., or *Anaplasma* spp. These findings underscore the importance of multiple sample types in assessing bats as reservoirs for zoonotic pathogens, particularly highlighting their role in transmitting pathogens through various body habitats, including saliva, urine, and ocular excretions. This study highlights the relevance of monitoring bat populations and studying their microbiota to enhance protections for both human and animal health.

## Simple Summary

Bats play a vital role in our ecosystems and can carry a variety of microorganisms, some of which may cause diseases in humans. As human activities alter their natural habitats, interactions between bats and people are increasing, making it crucial to understand the pathogens they harbor. This study focused on identifying potentially harmful bacteria and piroplasmids in bats from four caves in southern Italy. Over a span of more than two years, we collected samples from 149 bats representing different species. Our findings revealed the presence of *Bartonella henselae*, *Chlamydia* spp., and Piroplasmids in these bats and their ectoparasites, indicating that they could serve as sources of infection for both animals and humans. Bats were negative for the other investigated pathogens, including *Coxiella burnetii*, *Leptospira* spp., *Rickettsia* spp., *Anaplasma* spp., and *Borrelia* spp. Understanding the specific pathogens present in bats and their modes of transmission is essential for public health. This study underscores the importance of monitoring bat populations, as they can contribute to the spread of diseases, and highlights the need to study their microbiota to enhance protections for both human and animal health.

## 1. Introduction

Bats have attracted increasing attention in infectious disease research due to their role as natural reservoirs and sources of infection for a variety of microorganisms, including zoonotic pathogens capable of causing severe human diseases [1,2]. Bats inhabit a wide variety of ecological niches across most of the globe, with the exception of polar regions. Their habitats encompass a range of environments, including deserts, forests, and urban areas, with caves, bridges, and buildings serving as their main roosting sites. Owing to their distinctive adaptations and life history characteristics, bats play a crucial role in numerous ecosystems. Among their key adaptations are flight, which allows for migrations exceeding 2000 km, remarkable longevity (over 30 years) relative to other similarly sized mammals, and the ability to enter daily torpor or undergo seasonal hibernation. As primary nocturnal aerial predators, bats are essential for natural pest control in agricultural landscapes. Additionally, they contribute to reforestation and agricultural productivity through seed dispersal and pollination. With over 1400 identified species, bats represent nearly 20% of mammalian diversity. In Europe, 47 species have been documented [3]. These mammals are widespread, particularly in urban areas, where they frequently come into close contact with humans and domestic animals. Concurrently, human activities, such as urbanization and agricultural expansion, have led to the invasion of bat habitats, further increasing the risk of zoonotic pathogen spillover [4]. The unique ecological characteristics of bats, including their communal roosting behavior and exceptional longevity, play a critical role in the maintenance and transmission of microorganisms within bat colonies and to humans, either directly or indirectly [1,5].

Despite the growing recognition of bats as reservoirs for infectious agents, their role as carriers of bacterial pathogens has received considerably less attention compared to their involvement in viral epidemics [6,7]. Although a number of zoonotic bacterial pathogens have been detected in bats and their ectoparasites globally, research focusing on the European continent remains limited [8,9]. Nonetheless, evidence of bacterial transmission from bats to humans has been documented in certain cases, underscoring the need for more in-depth studies to fully understand the health risks posed by these pathogens [10,11].

Pathogens associated with bats can be transmitted to humans and animals through various transmission routes. These include direct contact with bodily fluids such as saliva, feces, or blood as well as transmission via vectors [12]. Notable examples of pathogens spread through environmental contamination are *Coxiella burnetii* and *Leptospira* spp. *Coxiella burnetii*, the agent responsible for Q fever, is a globally distributed zoonotic pathogen primarily transmitted through the inhalation of aerosols containing secretions from infected animals. Its global seroprevalence in humans varies widely, ranging from 2% to 30%, depending on geographic location, occupational exposure, and contact with livestock [13,14]. Similarly, Leptospirosis, caused by *Leptospira* spp., is a widespread zoonotic disease primarily transmitted through exposure to water or soil contaminated with the urine of infected hosts. It is particularly prevalent in tropical and subtropical regions, with an estimated 1.03 million human cases and 60,000 deaths annually worldwide [15].

Bats host a diverse range of arthropod ectoparasites [16] belonging to different families [17,18]. Some of them, such as Streblidae (Order: Diptera), Nycteribiidae (Order: Diptera), Ischnopsyllidae (Order: Siphonaptera), and Spinturnicidae (Order: Mesostigmata), are bat-specific, with the majority of their life cycle occurring on the bat’s fur or wing membranes [19,20].

Other bat ectoparasites are facultative, including species from the families Argasidae, (Order: Ixodida), Ixodidae (Order: Ixodida), Trombiculidae (Order: Trombidiformes), and Cimicidae (Order: Hemiptera), as they can parasitize other mammals and often spend extended periods away from bats [21]. These bat ectoparasites are frequently associated with a wide array of viruses [16,22,23,24,25]. Other kinds of pathogens have been less investigated in bat ectoparasites. Vector-borne pathogens transmitted by hematophagous arthropods therefore represent a matter of growing interest. Various members of the Chlamydiae phylum appear capable of colonizing bats, and their ectoparasites could represent promising candidates as vectors for the transmission of these bacteria. Chlamydiae are obligate intracellular bacteria responsible for infectious diseases with significant medical and economic impact. The family Chlamydiaceae includes two well-known human pathogens, *Chlamydia trachomatis* and *C. pneumoniae* as well as zoonotic species like *C. psittaci* and *C. abortus*, which primarily infect animals but can also pose risks to humans. These bacteria have been detected in diverse environments, including water and soil, and across a wide range of hosts, such as mammals, reptiles, birds, arthropods, and protozoans [26].

Among the vector-borne pathogens, transmitted particularly by ticks are *Anaplasma* spp., *Bartonella* spp., *Borrelia* spp., *Rickettsia* spp., and protozoan parasites such as *Babesia* and *Theileria* [27,28]. These are among the most widespread blood parasites globally and have a significant impact on human, veterinary, and ecological health. Over 60% of emerging human infectious diseases are zoonotic, with 71.8% originating from wildlife and 22.8% from arthropod vectors. The frequency of emerging vector-borne zoonoses has increased in the past decade, with tick-borne infections in humans primarily originating from wildlife [29].

Given the growing body of evidence regarding bats as reservoirs of various pathogens, this study aimed to evaluate the presence of a panel of zoonotic bacterial and protozoan pathogens in bats sampled from four caves in Sicily (Southern Italy).

Using molecular biology techniques, different samples (urine, fecal swabs, ocular conjunctival swabs and oropharyngeal swabs, ectoparasites) have been analyzed from these bats as well as guano samples from the caves to determine the potential presence of pathogens such as *C. burnetii*, *Leptospira* spp., *Anaplasma* spp., *Bartonella henselae*, *Borrelia* spp., *Rickettsia* spp., and Piroplasmids (*Babesia* and *Theileria*), contributing to the understanding of the circulation of these pathogens among bats and the health risks associated with bat-borne pathogens in this region.

## 2. Materials and Methods

### 2.1. Study Area

**Figure 1:**
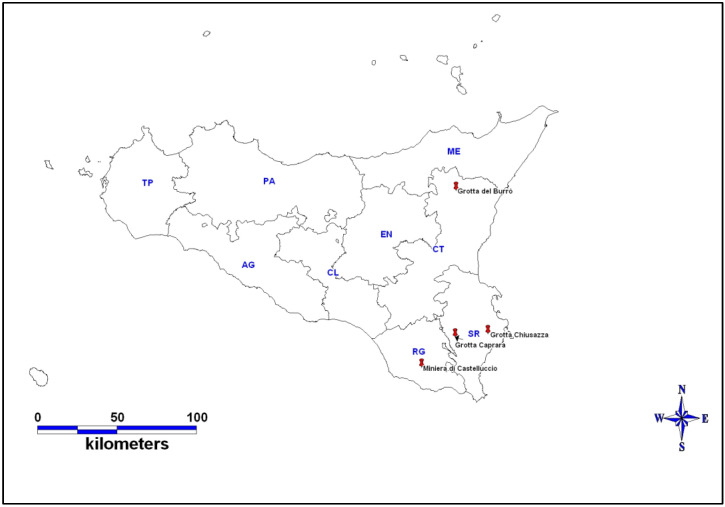
The geographical distribution of the four caves in provinces of Ragusa, Catania, and Syracuse in Sicily. The image was generated using MapInfo Pro (version 17.0).

The sampling was carried out in four different caves in provinces of Ragusa, Catania, and Syracuse in Sicily (Southern Italy) (Figure 1).

Grotta Chiusazza (Floridia, Syracuse; Lon 15.159536; Lat 37.026406) opens at an elevation of 107 m a.s.l. on the eastern margin of the Iblean plateau, within a karstic carbonate surface. The cave features two entrances located to the northwest and southeast, respectively. Proceeding from the southeast entrance, there is an initial stepped chamber. The total length of the cave is approximately 250 m, with a negative elevation difference of about 15 m. The area in which the cave opens is characterized by the presence of intensive monocultures and arable land and small ponds, fed by the runoff from farmland, where the bats go to feed and drink. The cave is populated by a large colony of *Minioptera* and dozens of Rhinolophids.

Grotta del Burrò (Randazzo, Catania; Lon 14.934538; Lat 37.826880) is a wide lava flow gallery that extends for approximately 250 m within prehistoric lava of uncertain age (15 Ka—3930 ± 60 a), with the eruptive source remaining unidentified. It has an overall elevation difference of about 47 m. The entrance is located within a large funnel-shaped depression. The surrounding area has a dense shrub–herbaceous vegetation cover and no arable land. In the territory, semi-wild cattle breeding is commonly practiced. The cave is inhabited by a large mixed colony of bats (about 600–700 *Minioptera* along with several dozen large *Myotis* and Rhinolophids).

Grotta Caprara (Noto, Syracuse; Lon 14.926690; Lat 37.007520) is aligned with other openings on the slightly terraced wall of an enormous bank that extends from northwest to southeast in a rather winding manner, rising approximately 200 m from the valley floor. The opening is quite wide and extends horizontally with minimal elevation change for nearly a hundred meters, but it follows a tortuous path. The cave is populated by a large colony (several hundred) of *Minioptera* and Rhinolophids.

Miniere di Castelluccio (Modica, Ragusa; Lon 14.691320; Lat 36.837110) is located within a much larger mining area situated on the terraces overlooking the course of the Irminio River. The area includes the open-pit mines of Castelluccio, where the level of mineralization is superficial. These are former mines used for the extraction of bituminous limestone (asphalt) until the mid-20th century. Their formation is presumably due to the infiltration of existing petroleum deposits in the subsurface, which migrated from depth along the paths of least resistance provided by discontinuity systems and deep fractures throughout the rock mass. The bat colony consists of approximately 200 Rhinolophids found within an underground quarry that is nearly flat, with a length of about 300 m and a maximum width of approximately 150 m. The presence of bats from other families has not been confirmed.

### 2.2. Sample Collection

The capture of bats was carried out only by expert and authorized personnel of the Università degli Studi di Catania (RG and MTS), according to current legislation. When possible, the bats were caught by hand in order to minimize the risk of disturbance to the colony. In some cavities a hand-held net with a telescopic handle was used to capture static and non-flying bats. After capture, each bat was placed for a short time inside a numbered canvas bag whose mouth was closed by a cord. Each bag contained only one subject. All operations, including the safety devices used by the operators, complied with the Guidelines for the monitoring of Chiroptera drawn up by the Istituto Nazionale Fauna Selvatica.

When possible, the following basic set of samples were collected from each bat: saliva (oropharyngeal swab), feces (fresh fecal sample or rectal swab), urine (urogenital swab), ocular-conjunctival swabs. The swabs were collected by using individually packed sterile microbiological swabs moistened with sterile saline solution 0.9%, inserting the tip and gently rotating it against the mucosa. The swabs were subsequently inserted into tubes containing a transport medium. Trapped bags were also inspected for the presence of ectoparasites, and when present, they were carefully removed using forceps, collected, and preserved in vials containing 70% ethanol for long-term storage. Each vial was labeled with the identification code of the corresponding sampled animal. Moreover, guano from the 4 caves was collected. All samples were stored at −20 °C, then transported to laboratory and stored at −80 °C until laboratory analysis.

The major bat species at the collection sites were determined based on morphological identification carried out by field experts (RG and MTS) and on previous data from bats’ roosting sites. These species were *Rhinolophus ferrumequinum*, *Rhinolophus hipposideros*, *Rhinolophus euryale*, *Myotis myotis*, and *Myotis capaccinii, Miniopterus schreibersii*.

All bats were handled for the shortest time possible and were released immediately after sampling.

### 2.3. Bat Ectoparasites Morphological Identification

Bat ectoparasite species were first identified based on key morphological traits, including the presence or absence of wings, using a dissecting microscope [30]. The collected Diptera were identified following Theodor’s identification keys [31]. Spinturnicid mites were identified based on the references provided by Rudnick (1960) [32], Stanyukovich (1997) [33], and Uchikawa and Kobayashi (1978) [34]. Ixodidae mites were identified according to their morphological characteristics, using the identification keys of Manilla (1998) [35].

### 2.4. DNA Extraction from Bat Samples

DNA was extracted from bat samples, guano, and ectoparasites by DNeasy blood and tissue kit (Qiagen, Germany) following the manufacturer’s protocol. Aliquots of extracted DNA were quantified using NanoDrop™ 2000 spectrophotometer and then stored at −20 °C until further use.

### 2.5. Bat Ectoparasites Molecular Identification

To confirm ectoparasites identification, at least at the genus level, we analyzed the *cytochrome c oxidase subunit I* (*COI*) gene, which is approximately 710 base pairs long. This gene was amplified via polymerase chain reaction (PCR) using primers specific to invertebrates [36].

### 2.6. Molecular Detection of Bacteria and Piroplasmids

The relevant genes targeted were *OmpA* and *OmpB*, *16S–23S internal transcribed spacer* (*ITS*), *insertion sequence* (*IS1111*), *32-kDa lipoprotein* (*lipL32*) gene, *OspA*, *23S rRNA*, *16S rRNA* and *18S rRNA* gene for *Rickettsia* spp., *Bartonella henselae*, *Coxiella burnetii*, *Leptospira* spp., *Borrelia* spp., *Chlamydia* spp., *Anaplasma* spp., and Piroplasmids, respectively. The reactions for *Rickettsia* and *Anaplasma* were run under nested PCR/PCR settings; the other reactions were run under the real-time PCR settings, following protocols published elsewhere [37,38,39,40,41,42,43,44,45,46]. All primers and probes used in this study are listed in Table 1.

Real-time PCR assays carried out in a CFX96 Real-Time System (Bio-Rad, Hercules, CA, USA) or QuantStudio™ 6 Pro Real-Time PCR System (Life Technologies, Thermo Fisher Scientific, Carlsbad, CA, USA). The reaction mix included 1X SsoAdvanced Universal Probes Supermix (Bio Rad), 500 nM of each primer and 500 nM of probe, in a 20 µL total volume. The following thermal cycle conditions were used: 95 °C for 3 min and 40 cycles of 95 °C for 10 s and 60 °C for 30 s.

Conventional PCR assays were carried out in a SimpliAmp thermal cycler (Thermo Fisher Scientific Inc., Waltham, MA, USA) in a final volume of 50 µL, using GoTaq G2 DNA Polymerase (Promega Italia s.r.l., Milan, Italy) with 5 µL of each DNA extract. Positive and negative controls were included in each amplification assay to evaluate the presence of appropriately sized amplicons and to rule out potential contamination, respectively. The amplicons were visualized by electrophoresis on a 2% agarose gel and observed using a SybrSafe nucleic acid staining solution under UV light. A 100 bp DNA ladder was used as a molecular-weight size marker (Amplisize^®^ Molecular Ruler, BioRad Laboratories, CA, USA). The PCR products were quantified and sent for sequencing to Macrogen Inc. (Macrogen Europe, Amsterdam, the Netherlands). The analyses for tick-borne pathogens were carried out by the Italian National Reference Center for *Rickettsia, Anaplasma*, *Babesia*, and *Theileria* (C.R.A.Ba.R.T.).

### 2.7. Molecular Confirmation of Chlamydia spp.

*Chlamydia* spp. positive samples were sent to the Italian National Reference Centre for Chlamydiosis for confirmation and molecular characterization. In particular, five samples of DNAs were screened for Chlamydiaceae by a real-time PCR targeting *23S rRNA* gene [43] using a final concentration of 0.6 μM of each primer and 0.3 μM of probe. For the PCR reaction, the QuantiFast Pathogen PCR +IC Kit (Qiagen) was used with an internal control of amplification. In each run, positive and negative controls were also included. The amplification cycle consisted of 5 min at 95 °C, followed by 45 cycles of denaturation at 95 °C for 15 s and annealing and extension at 60 °C for 30 s. In order to identify the chlamydial species, positive samples were tested, first with a species-specific real time for *C. psittaci* [47] using the QuantiFast Pathogen PCR mastermix (Qiagen) with a final concentration of 0.6 μM of each primer and 0.25 μM of probe. The amplification cycles were the same used for screening real-time PCR. Then, the samples were tested with an end-point PCR targeting the *16S* ribosomal gene [48]. The PCR reaction mixture in a total volume of 25 µL consisted of 1X PCR GoTaq Flexi Buffer (Promega), 0.4 µM of each primer, 0.4 mM of deoxyribonucleotide triphosphate (dNTP), 2.5 mM of MgCl2, 1U of GoTaq Flexi DNA polymerase (Promega), and 5 µL of DNA sample. Amplification conditions for 16S rDNA were carried out with an initial denaturation at 94 °C for 2 min, followed by 10 cycles of denaturation at 94 °C for 30 s, annealing at 56 °C for 30 s, and extension at 72 °C for 30 s and 35 cycles of denaturation at 94 °C for 30 s, annealing at 52 °C for 30 s, and a final extension at 72 °C for 5 min. Amplicons were sequenced and blasted in NCBI Genbank. The *16S rDNA* gene sequences obtained in this study were employed for phylogenetic analyses, together with selected database sequences downloaded from NCBI. Sequences were aligned using MUSCLE software version 3.8 [49]. The best sequence evolution model was established using jModelTest software version 2 [50], and maximum likelihood (ML) phylogenetic trees were inferred using PhyML [51] with 100 bootstrap pseudo-replicates.

## 3. Results

### 3.1. Bat Sampling

From December 2020 to April 2023, 149 bats were captured from the four different caves. The animals belonged to six species of three genera, including *Myotis capaccinii* (*n* = 1), *Myotis myotis* (*n* = 2), *Miniopterus schreibersii* (*n* = 90), *Rhinolophus euryale* (*n* = 6), *R. ferrumequinum* (*n* = 43), and *R. hipposideros* (*n* = 7) (Table 2). *Miniopterus schreibersii* accounted for more than half of the tested bats (60%). Other bat species accounted for a smaller portion, ranging from 0.7% to 28.8%.

The number of bat species varied by site, with five, three, and two different species detected in Grotta del Burrò, Grotta Chiusazza, and Grotta Caprara, Miniera di Castelluccio, respectively (Table 3).

### 3.2. Morphological and Molecular Identification of Bat Ectoparasites

A total of 16 ectoparasites of bats have been identified. Specifically, from bats originating from the Grotta Chiusazza, nine *Penicillidia conspicua*, one *Nycteribia schmidlii*, and one *Phthiridium biarticulatum* were collected from *Miniopterus schreibersii*, while one *Penicillidia conspicua* and one *Ixodes vespertilionis* were collected from *Rhinolophus ferrumequinum*. Two *Phthiridium biarticulatum* and one *Ixodes vespertilionis* were collected from *Rhinolophus ferrumequinum* from the Miniera di Castelluccio. Morphological identification provided reliable results on the examined taxa. Additionally, molecular analyses were performed, which confirmed the results obtained through morphological investigation.

### 3.3. Molecular Detection of Pathogens

**Figure 2:**
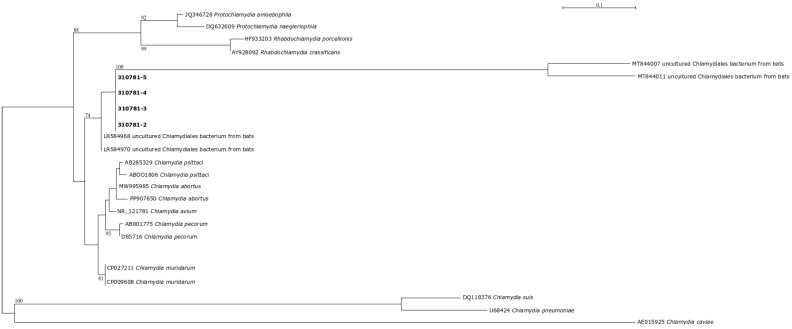
A phylogenetic tree constructed with the obtained and selected database sequences from NCBI. Alignments were performed using MUSCLE software (version 3.8). The best evolution model was established using jModelTest (version 2), and maximum likelihood (ML) phylogenetic trees were inferred using PhyML with 100 bootstrap pseudo-replicates.

*Chlamydia* spp. DNA was detected in a sample of guano from Grotta del Burrò, in feces, ocular conjunctival and oropharyngeal swabs of a *Miniopterus schreibersii* from Grotta Chiusazza, and in urine samples of four *Rhinolophus ferrumequinum* from Grotta Caprara. All positive samples were sent to the National Reference Center for *Chlamydia* and identified as *Chlamydia* spp. by real-time PCR. The presence of *Chlamydia psittaci* DNA was excluded by specific real-time PCR. In addition, Chlamydiaceae presence was demonstrated by PCR/RFLP, and the sequencing results showed a nucleotide identity of 96.95% with uncultured *Chlamydiales* bacterium. Phylogenetic analyses confirmed the association of the obtained sequences with uncultured *Chlamydiales* bacteria from bats (Figure 2).

*Bartonella henselae* DNA was detected in 3 of the 149 tested bats, giving the overall prevalence of 2%. *Bartonella* DNA was detected in both ocular conjunctival and oropharyngeal swabs of *Myotis capaccinii* from Grotta del Burrò and in oropharyngeal swabs of two *Miniopterus schreibersii* from Grotta Chiusazza.

Piroplasmids DNA was detected in 10 of 149 fecal swabs (6.7%), 6 of *Miniopterus schreibersii* and 4 of *Rhinolophus ferrumequinum* from Grotta Chiusazza. Additionally, DNA of Piroplasmids was also detected in 5 of 16 (31.2%) bat ectoparasites: specifically 3 *Penicillidia conspicua* collected from *Miniopterus schreibersii* and 1 *Ixodes vespertilionis* collected from *Rhinolophus ferrumequinum* of the Grotta Chiusazza and 1 *Ixodes vespertilionis* collected from *Rhinolophus ferrumequinum* of the Miniera di Castelluccio.

No samples tested positive for Leptospira, Borrelia, Coxiella, Rickettsia, and Anaplasma.

## 4. Discussion

In this study, 149 bats were collected from four different caves in Sicily. The animals were morphologically identified. Ectoparasites collected from bats were identified with morphological and molecular methods. Urine, fecal swabs, ocular conjunctival swabs, oropharyngeal swabs, and guano samples were examined for zoonotic agents through molecular methods. The study revealed the presence of *Bartonella henselae*, *Chlamydia* spp., in bats. Phylogenetic analyses confirmed the association of the obtained sequences with uncultured *Chlamydiales* bacteria from bats. Moreover Piroplasmid DNA was detected in bats and in their ectoparasites.

Currently, 26 species of insectivorous bats are present in Sicily. These bats play crucial ecological roles, particularly as natural predators of various pest insects and as bioindicators of environmental alterations. However, the rapid pace of anthropogenic and environmental changes—such as agricultural practices, habitat fragmentation, wind energy installations, and climate change—are considered the primary threats facing Mediterranean bats, including those on islands [52].

In particular, six species of bats have been documented in the geographical areas involved in this study, some of which are at risk of extinction.

*Myotis capaccinii* (Bonaparte, 1837) is a small bat characterized by gray fur that is lighter on the ventral side. This species is typically cavernicolous, favoring natural or artificial underground cavities for roosting throughout the year [53]. These roosts are ideally situated near rivers or bodies of water that have riparian vegetation, which are important for its feeding activities. *Myotis capaccinii* is found at elevations ranging from sea level up to 825 m. It is a species in significant decline and is poorly represented in Italy, with fewer than 20 known colonies, despite its strong affinity for Mediterranean habitats. Foraging occurs primarily in open areas, especially over water. This bat feeds on a variety of insects, particularly Trichoptera, Neuroptera, and Diptera, and it may also catch fish just above or just below the water surface. According to the IUCN Red List (2013) [54], *Myotis capaccinii* is classified as Endangered (EN) under criterion A2c.

*Myotis myotis* (Borkhausen, 1797) is a large bat species that thrives in warm climates. It prefers temperate and warm locations in lowland and hilly areas, inhabiting a variety of environments from sea level up to 600 m, although it can range as high as 2000 m [55]. Colonies of *Myotis myotis* are primarily established in deciduous or mixed forests that have sparse ground vegetation [56]. Foraging sites are characterized by easy access to open spaces, allowing the bats to hunt arthropods living on the ground. The majority of their prey consists of ground-dwelling arthropods that are longer than 1 cm, with a significant prevalence of Carabidae beetles. According to the IUCN Red List (2013) [54], *Myotis myotis* is classified as Least Concern (LC), indicating a stable demographic trend.

*Miniopterus schreibersii* (Kuhl, 1817) is a medium-sized bat characterized by a slender body, a short snout, and widely spaced triangular ears. This species is typically cavernicolous, predominantly associated with environments that are either uninhabited or lightly anthropized, with a particular preference for karst habitats. It favors areas of low to medium altitude, ranging from coastal regions to mountainous areas. *Miniopterus schreibersii* is fundamentally a sedentary species, especially in the southern regions where the climate is milder. They forage in flight, preying on various species of small insects. Their diet mainly consists of Lepidoptera, although they also consume small percentages of Neuroptera, Diptera, and small beetles. According to the IUCN Red List (2013) [54], *Miniopterus schreibersii* is classified as Near Threatened (NT), indicating a declining demographic trend.

*Rhinolophus euryale* (Blasius, 1853) is a medium-sized bat that can be easily distinguished from its congeners by examining the shape of its noseleaf, which features a sharply pointed and forward-curving saddle. This species is found throughout Italy, with the exception of certain areas in the northern regions. It has been reported at elevations from sea level up to approximately 1000 m, although it prefers lower altitudes as it is a thermophilic species. *Rhinolophus euryale* inhabits Mediterranean environments that exhibit karstic features and are characterized by abundant forest or shrub cover. In these habitats, it hunts flying arthropods both in flight and from ambush. According to the IUCN Red List (2013) [54], *Rhinolophus euryale* is classified as Vulnerable (VU) under criterion A2c.

*Rhinolophus ferrumequinum* (Schreber, 1774) is the largest species of rhinolophid found in Europe, with a forearm measuring 54–61 mm and a weight that often exceeds 20 g. It is present throughout Italy and has been reported from sea level up to 2000 m, although it prefers areas below 800 m, particularly in climate-mild stations characterized by vegetation mosaics (e.g., pastures interspersed with hedges and deciduous forest formations) and the presence of wetlands. Day roosting sites, breeding, and hibernation are typically found in underground cavities and buildings. The diet primarily consists of large insects, which are captured in flight at low heights or, less frequently, while on the ground [57]. According to the IUCN Red List (2013) [54], *Rhinolophus ferrumequinum* is classified as Vulnerable (VU) under criterion A2c.

*Rhinolophus hipposideros* (Bechstein, 1800) is a small and delicate-looking bat that can be distinguished from its congeners by its tiny size (when hanging, it is about the length of a thumb). The forearm measures 35–40 mm, and the weight ranges from 4 to 6 g. This species is found throughout Italy, as well as in all European countries, including islands; across the Mediterranean basin; and in North and East Africa and the Sinai [58]. It has been reported from sea level up to 2000 m in altitude. Ideal foraging sites include deciduous forests or areas characterized by a mix of forest patches, open spaces, and wetlands. Day roosting, breeding, and hibernation sites are typically found in underground cavities. The diet consists mainly of small insects and spiders, with prey being captured in flight or while resting on vegetation or the ground [57]. According to the IUCN Red List (2013) [54], *Rhinolophus hipposideros* is classified as Endangered (EN) under criterion A2c.

Among ectoparasites, three species of bat flies, *Penicillidia conspicua*, *Phthiridium biarticulatum*, and *Nycteribia schmidlii*, and a species of bat tick, *Ixodes vespertilionis*, were identified.

Bat flies (Diptera: Nycteribiidae, and Streblidae) are usually the most abundant ectoparasites of bats, mostly wingless insects that live in the fur and on the wing membranes, feeding on blood. They infest host species very specifically. Bats can be reservoirs for numerous zoonotic diseases that can affect humans, livestock, or companion animals, some of which are vector borne. Bat flies are vectors of several pathogens (some even zoonotic), including bacteria such as *Bartonella* spp., piroplasmids, and trypanosomes. The epidemiological importance of a competent vector is directly related to its ability to infest as many new hosts as possible (including different host species), which is why host specificity is a key trait of insect vectors. High host specificity develops in a parasite to increase its ability to exploit a particular susceptible host, and it typically results from long-term host–parasite co-speciation. Since host specificity is a determining factor influencing pathogen circulation in host–parasite systems, knowledge of bat fly host preferences can provide insight into the likelihood of pathogen spread by these vectors. Our understanding of bat ectoparasites in Europe is limited, although it has been shown that bat flies can have an ecological impact on their hosts and may be vectors of zoonotic diseases [59].

In our study, the bat fly *Penicillidia conspicua* was collected on *Miniopterus schreibersii* and *Rhinolophus ferrumequinum.* The bat fly *Penicillidia conspicua* is a hematophagous, obligate ectoparasite, highly specialized to their bat hosts. The geographical distribution of *Penicillidia conspicua* reflects that of its host, ranging from the Mediterranean region in Europe to northern Africa and the Middle East. *Penicillidia* spp. are vectors of batmalaria, *Polychromophilus* spp., and suspected vectors of the bacteria group *Bartonella*. It has also been found to host other pathogens, such as rhabdoviruses [60].

Our investigation reported the presence of *Phthiridium biarticulatum* on *Rhinolophus ferrumequinum*, confirming that *R. ferrumequinum* is one of the primary hosts of this fly species [30].

*Nycteribia schmidlii* was found in association with *Miniopterus schreibersii*, as reported in several European regions, including Italy [30].

In our study, the bat-associated tick *Ixodes vespertilionis* was collected on *Rhinolophus ferrumequinum*, confirming previous observations that it predominates on *Rhinolophus* spp. [61]. *Ixodes vespertilionis* has a worldwide distribution and has been recorded on many species of Rhinolophidae and Vespertilionidae [61]. Bat-associated ticks have been found to harbor a wide range of pathogens, some of which are of significant veterinary and public health relevance, including species from the following genera: *Anaplasma*, *Bartonella, Borrelia*, *Mycoplasma*, *Rickettsia*, and *Babesia* [8,62,63,64,65,66,67]. Despite their importance, ubiquity, and relevance to human health, the potential transmission routes of these pathogens from bats remain poorly understood. Human pathogenic *Borrelia* species have only recently been detected for the first time in bat ticks [67]. Bat-associated *Borrelia* spp. can occasionally infect humans as well [68]. Furthermore, *Bartonella* species and antibodies associated with bats have also been observed in humans in Europe, suggesting their potential to cause disease [69,70]. Host specificity can vary significantly among ticks, which can influence their role in disease transmission [71]. Bat-associated ticks exhibit different levels of host specificity depending on the species [72].

Previous studies on bacterial pathogens associated with bats in the caves under investigation have primarily focused on the intestinal microbiome. These studies have employed microbiological culture techniques and detailed analyses related to antibiotic resistance to characterize microbial diversity and the presence of resistant strains [73,74]. However, to date, there is a lack of evidence regarding specific research on the pathogens considered in the present study, particularly those found in samples collected from other body regions of the bats, such as urine, oropharyngeal swabs, and conjunctival swabs. This knowledge gap limits our understanding of the ecological role that bats may play in the transmission of pathogens, especially zoonotic agents, which can have significant implications for public health. The scarcity of data on these aspects hinders our ability to assess the risks associated with disease transmission and biodiversity conservation.

Analyzing samples collected from various body sources could provide critical information on the prevalence, distribution, and behavior of pathogens, thereby contributing to a more comprehensive understanding of the health of the ecosystems in which these bats operate. An integrated approach that includes sampling from different body parts and the use of advanced microbiological analysis methodologies may prove essential for delineating a more complex picture of the ecological and health interactions characterizing these organisms and their habitats. Furthermore, such investigations could facilitate the development of more effective monitoring and conservation strategies to mitigate the risks associated with the spread of zoonotic pathogens.

One of the most significant findings of our study is the detection of *Chlamydia* spp. in samples of guano, feces, ocular conjunctival and oropharyngeal swabs, and urine collected from *Miniopterus schreibersii* and *Rhinolophus ferrumequinum*.

*Chlamydiae* are obligate intracellular bacteria and are typical pathogens to both humans and animals, but they cannot survive outside their hosts [75].

Our findings confirm the association between *Chlamydia* and bats, as reported in several published scientific works. In a study carried out in the urban wildlife of Madrid city (Spain) *Chlamydia* was detected in bat fecal samples, and bats appeared to be a species of great importance as a reservoir of zoonotic organisms [76]. Chlamydiae were among the most dominant phyla found in bats droppings in a study carried out in Cambodia [75]. A recent study found that more than 50% of the fecal samples of the bat *Myotis daubentonii* (Chiroptera: Vespertilionidae) carried members of the Chlamydiae phylum in Finland, with most of the positive samples belonging to two main families, the Rhabdochlamydiaceae and the Chlamydiaceae [26]. Although bats seem to be colonized by various members of the Chlamydiae phylum, whether these strict intracellular bacteria are transmitted between bats is yet unknown. However, ectoparasites of bats may be suitable vector candidates for Chlamydiae bacteria. Unlike a previous study, which reported a high prevalence (57.95%) of *Chlamydiae* in *Spinturnix myoti*, an obligate ectoparasitic mite species of mouse-eared bats (*Myotis* spp.), suggesting that mites may act as reservoirs or vectors for this pathogen [26], in our study, no *Chlamydia* spp. were detected in ectoparasites collected from bats, likely due to the small sample size of the latter.

*Bartonella* DNA was detected in ocular conjunctival and oropharyngeal swabs of two species of bat: *Myotis capaccinii* and *Miniopterus schreibersii*.

In a study conducted as a quantitative metagenomic analysis of the fecal bacterial flora of the Daubenton’s bat in Finland, DNA of *Bartonella* spp. was either detected or isolated from the peripheral blood bats and detected in the ectoparasites of bats. The blood isolates belong to the Candidatus species *Bartonella mayotimonensis*, identified as an etiologic agent of endocarditis in humans, and a new *Bartonella* species (*Bartonella naantaliensis* sp. nov.). A phylogenetic analysis of bat-colonizing *Bartonella* spp. throughout the world demonstrates a distinct *Bartonella mayotimonensis* cluster in the Northern Hemisphere. The findings of this field study highlight bats as potent reservoirs of human bacterial pathogens [5].

Similarly, *Bartonella* spp., which can cause febrile illnesses and endocarditis in humans, have been isolated from bats and their ectoparasites in several countries. The presence of hemoparasites from the *Bartonella* genus was detected in DNA extracted from the macerated heart tissue of dead bats in southwest England [6]. Another study conducted in Ukraine detected *Bartonella* spp. DNA in 12.1% of bat ectoparasites, particularly in fleas and bat flies [9].

Although the risk of human infection through these vector-borne pathogens is still being assessed, their presence in bat-associated ectoparasites suggests a potential role for bats in the ecological cycle of these diseases. In addition to bacterial pathogens, bats also harbor protozoan parasites such as *Babesia* and *Theileria*, which are transmitted by ticks and are emerging zoonotic pathogens. In this study, Piroplasmid DNA was detected in a total of 10 fecal swabs collected from *Miniopterus schreibersii* and *Rhinolophus ferrumequinum* and in five bat ectoparasites of two species: *Penicillidia conspicua* and *Ixodes vespertilionis. Babesia vesperuginis* was first reported in bats in Italy in 1898, and since then, various species of *Babesia* and *Theileria* have been detected in bat-specific ticks, such as *Ixodes ariadnae*, *Ixodes vespertilionis*, and *Ixodes simplex*, in European countries like Hungary and Romania [27,63]. DNA sequences of piroplasms were detected in three bat ticks (2.5%) belonging to the species *Argas vespertilionis* in the United Kingdom [62]. These protozoan parasites, which are among the most widespread blood parasites worldwide, have significant implications for human, veterinary, and ecological health.

Although not detected in this study, bacterial pathogens transmitted by hematophagous vectors, particularly ticks, remain a significant concern. For example, *Anaplasma* spp., responsible for anaplasmosis, have been detected in bat ectoparasites in Europe, with *Anaplasma phagocytophilum* identified in *Ixodes ariadnae*, a bat-specific tick species in Hungary [8].

*Borrelia* spp., the causative agents of Lyme disease, have also been identified in ticks associated with bats in countries such as Hungary and Spain [7,8].

Furthermore, *Rickettsia* spp., responsible for spotted fever group rickettsioses, have been found in bat ectoparasites, particularly in Spain and Hungary [8].

No DNA of *Leptospira* spp. or *Coxiella burnetii* was detected in the bats examined in this study. While traditionally associated with livestock, *C. burnetii* has been detected in bats and their ectoparasites in Europe, such as in Spain, where DNA of this pathogen was found in bat feces and guano, suggesting potential environmental contamination [77]. In Algeria, *C. burnetii* has also been identified in bat ectoparasites, reinforcing the hypothesis that bats may serve as alternative reservoirs of this zoonotic pathogen [78].

European bats are known to act as carriers or reservoirs of various zoonotic bacterial pathogens, including *Leptospira* spp. Through the use of diverse methodologies, *Leptospira* spp. have been identified in over 50 bat species from various geographic regions, predominantly in tropical and subtropical areas. However, the role of bats in the epidemiology of leptospirosis in Europe remains largely undefined, as the occurrence of *Leptospira* spp. in European bat populations is still poorly documented.

*Leptospira* species have been reported in bat populations across Europe, including in countries such as France, Denmark, the Czech Republic, and Poland [79,80].

Overall, bats appear to be a species of great importance as a reservoir of zoonotic organisms. As the methodology used does not allow us to be sure that the detected DNA comes from viable cells, in future studies, it would be very important to carry out specific monitoring of the pathogens present in bat populations.

## 5. Conclusions

This study provides valuable insights into the diversity of bat species, their ectoparasites, and the presence of zoonotic pathogens in various cave ecosystems from Southern Italy. Among the 149 bats captured, *Miniopterus schreibersii* represented the majority, with five other species identified across three genera. The species distribution varied between trapping sites, highlighting distinct ecological niches.

Morphological and molecular analyses successfully identified four ectoparasite species, including bat flies (*Penicillidia conspicua*, *Nycteribia schmidlii*, and *Phthiridium biarticulatum*) and the tick *Ixodes vespertilionis*. The combined methodological approach ensured the accurate taxonomic identification of both hosts and their ectoparasites.

Pathogen screening revealed the presence of *Chlamydia* spp. DNA in multiple samples from different sites and species, with a phylogenetic analysis associating the sequences with uncultured Chlamydiales bacteria found in bats. The study also identified a low prevalence of *Bartonella henselae* DNA (2%) and a high prevalence of Piroplasmids in bats (21.8%) and in bat ectoparasites (31.2%).

Importantly, no evidence of other zoonotic pathogens, such as *Leptospira*, *Borrelia*, *Coxiella*, *Rickettsia*, or *Anaplasma*, was detected in the tested samples.

Bat-associated bacterial pathogens, transmitted through direct contact with infected bats or their ectoparasites (e.g., ticks), pose a potential risk to human health. However, spillover events remain rare or inadequately documented due to insufficient surveillance. Further research on pathogen diversity, seasonality, and bats’ role as reservoirs is critical to understanding disease transmission dynamics. Enhanced surveillance is needed to prevent zoonotic spillover and safeguard host populations. Incorporating these findings into public health and conservation policies is essential for effective pathogen management and biodiversity preservation.

## Figures and Tables

**Table 1:** PCR performed for the amplification of different *Rickettsia* spp. molecular targets and *Anaplasma* spp. and the real-time PCRs performed for the amplification of *Coxiella burnetii* and *Borrelia* spp., *Bartonella henselae*, *Chlamydia* spp., *Leptospira* spp., and Piroplasmids.

Pathogen	Gene Target	PCR Assay	Primer/Probe Sequences	Reference
*Rickettsia* spp.	*OmpA*	Nested PCR	Rr190.70p 5′-ATGGCGAATATTTCTCCAAAA-3′ Rr190.701n 5′-GTTCCGTTAATGGCAGCATCT-3′ Rr190.602n 5′-AGTGCAGCATTCGCTCCCCCT-3′	[37]
*Rickettsia* spp.	*OmpB*	Nested PCR	rompB OF 5′-GTAACCGGAAGTAATCGTTTCGTAA-3′ rompB OR 5′-GCTTTATAACCAGCTAAACCACC-3′ rompB SFG IF 5′-GTTTAATACGTGCTGCTAACCAA-3′ rompB SFG IR 5′-GGTTTGGCCCATATACCATAAG-3′	[38]
*Anaplasma* spp.	*16S-rRNA*	Nested PCR	EE1 5′-TCCTGGCTCAGAACGAACGCTGGCGGC-3′ EE2 5′-AGTCACTGACCCAACCTTAAATGGCTG-3′ EE3 5′-GTCGAACGGATTATTCTTTATAGCTTGC-3′ EE4 5′-CCCTTCCGTTAAGAAGGATCTAATCTCC-3′	[39]
*Coxiella burnetii*	*IS1111*	Real-time	sIS1pri F 5′-CGGGTTAAGCGTGCTCAGTAT-3′ sIS1pri R 5′-TCCACACGCTTCCATCACCAC-3′ Tqpro sIS1 (5′-FAM/3′-BHQ1) 5′-AGCCCACCTTAAGACTGGCTACGGTGGAT-3′	[40]
*Borrelia* spp.	*OspA*	Real-time	Bor_OspA_F 5′-AATATTTATTGGGAATAGGTCTAA-3′ Bor_OspA_R 5′-CACCAGGCAAATCTACTGA-3′ Bor_OspA_TM (5′-FAM/3′-BHQ1) 5′-TTAATAGCATGYAAGCAAAATGTTAGCA-3′	[41]
*Bartonella henselae*	*16S-23S rRNA ITS*	Real-time	B_hen F 5′-TAGTGAACGGGATTAGATAC-3′ B_hen R 5′-CAAAACAAAGTGCAAAACAA-3′	[42]
*Chlamydia* spp.	*16S–23S rRNA*	Real-time	Ch23S-F 5′-CTGAAACCAGTAGCTTATAAGCGGT-3′ Ch23S-R 5′-ACCTCGCCGTTTAACTTAACTCC-3′ Ch23S-P (5′-FAM/3′-TAMRA) 5′-CTCATCATGCAAAAGGCACGCCG-3′	[43]
*Leptospira* spp.	*Lip32* *16S rRNA*	Real-time	LipL32-45F 5′-AAG CAT TAC CGC TTG TGG TG-3′ LipL32-45R 5′-GAACTCCCATTTCAGCGATT-3′ LipL32-189P Probe (5′-FAM/3′-BHQ1) 5′-AAAGCCAGGACAAGCGCCG-3′ Lep-F 5′-TAGTGAACGGGATTAGATAC-3′ Lep-R 5′-GGTCTACTTAATCCGTTAGG-3′ Lep-Probe (5′-FAM/3′-BHQ1) 5′-AATCCACGCCCTAACGTTGTCTAC-3′	[44,45]
Piroplasmids	*18S rRNA*	Real-time	Bab18S_F 5′-CATGAACGAGGAATGCCTAGTATG-3′ Bab18S_R 5′-CCGAATAATTCACCGGATCACTC-3′ Bab18S_Pr (5′-FAM/3′-BQ1) 5′-CCGAATAATTCACCGGATCACTC-3′	[46]

**Table 2:** The bats sampled in the study.

Superfamily	Family	Species	n. Bats (%)
Vespertilionoidea	Vespertilionidae	*Myotis capaccinii*	1 (0.7%)
		*Myotis myotis*	2 (1.3%)
	Miniopteridae	*Miniopterus schreibersii*	90 (60.4%)
Rhinolophoidea	Rhinolophidae	*Rhinolophus euryale*	6 (4.0%)
		*Rhinolophus ferrumequinum*	43 (28.9%)
		*Rhinolophus hipposideros*	7 (4.7%)
Total			149

**Table 3:** The geographic distribution of the sampled bats.

Bat Species	Grotta del Burrò	Grotta Chiusazza	Miniera di Castelluccio	Grotta Caprara
*Myotis capaccinii*	1	/	/	/
*Myotis myotis*	2	/	/	/
*Miniopterus schreibersii*	12	55	/	23
*Rhinolophus euryale*	4	/	/	2
*Rhinolophus ferrumequinum*	1	20	21	1
*Rhinolophus hipposideros*	/	4	3	/
Total	20	79	24	26

## Data Availability

Data are contained within the article.
